# 

**Published:** 1886-12

**Authors:** 


[NOTE.—Owing to the length of some of the papers in this issue we have been obliged to omit all Editorial, and Book notices, as well as advertisers' notices.—Ed.]

